# The effect of vitamin D, magnesium and zinc supplements on interferon signaling pathways and their relationship to control SARS-CoV-2 infection

**DOI:** 10.1186/s12948-021-00161-w

**Published:** 2021-11-08

**Authors:** Mohsen Nabi-Afjadi, Hadis Karami, Kaveh Goudarzi, Iraj Alipourfard, Elham Bahreini

**Affiliations:** 1 Department of Biochemistry, Faculty of Biological Science, Tarbiat Modares University, Tehran, Iran; 2 Department of Molecular Cell Biology and Microbiology, Faculty of Biological Science and Technology, University of Isfahan, Isfahan, Iran; 3 Nursing Department, Islamic Azad University, Khorasgan Branch, Isfahan, Iran; 4 Institute of Biology, Biotechnology and Environmental Protection, Faculty of Natural Sciences, University of Silesia, Bankowa 9, 40-007 Katowice, Poland; 5 Department of Biochemistry, Faculty of Medicine, Iran University of Medical Sciences, P.O. Box: 1449614525, Tehran, Iran

**Keywords:** COVID-19, Interferon, Vitamin D, Magnesium, Zinc

## Abstract

The concern of today's communities is to find a way to prevent or treat COVID-19 and reduce its symptoms in the patients. However, the genetic mutations and more resistant strains of severe acute respiratory syndrome coronavirus 2 (SARS-CoV-2) emerge; the designed vaccines and adjuvant therapies would potentially control the symptoms and severity of COVID-19. The most important complication of this viral infection is acute respiratory distress syndrome, which occurs due to the infiltration of leukocytes into the alveoli and the raised cytokine storm. Interferons, as a cytokine family in the host, play an important role in the immune-related antiviral defense and have been considered in the treatment protocols of COVID-19. In addition, it has been indicated that some nutrients, including vitamin D, magnesium and zinc are essential in the modulation of the immune system and interferon (IFN) signaling pathway. Several recent studies have investigated the treatment effect of vitamin D on COVID-19 and reported the association between optimal levels of this vitamin and reduced disease risk. In the present study, the synergistic action of vitamin D, magnesium and zinc in IFN signaling is discussed as a treatment option for COVID-19 involvement.

## Introduction

Currently, much of the world's attention is focused on finding a way to prevent or treat COVID-19 disease or reduce its symptoms in patients. Many researchers have offered various solutions and recommendations in this regard, but they need to be proven. Severe acute respiratory syndrome coronavirus 2 (SARS-CoV-2) belongs to the positive-strand RNA viruses family with a crown-like appearance. The viral genome contains S, E, M, and N genes that encode structural proteins and the ORF region, which expresses non-structural proteins such as papain-like protease, 3-chymotrypsin-like protease and RNA-dependent RNA polymerase [1]. The virus spreads by the scattered droplets through coughing, sneezing, or exhaling from a person's mouth and nose with COVID-19. The virus enters the nasal system by inhalation and begins to multiply [2].

**Fig. 1:**
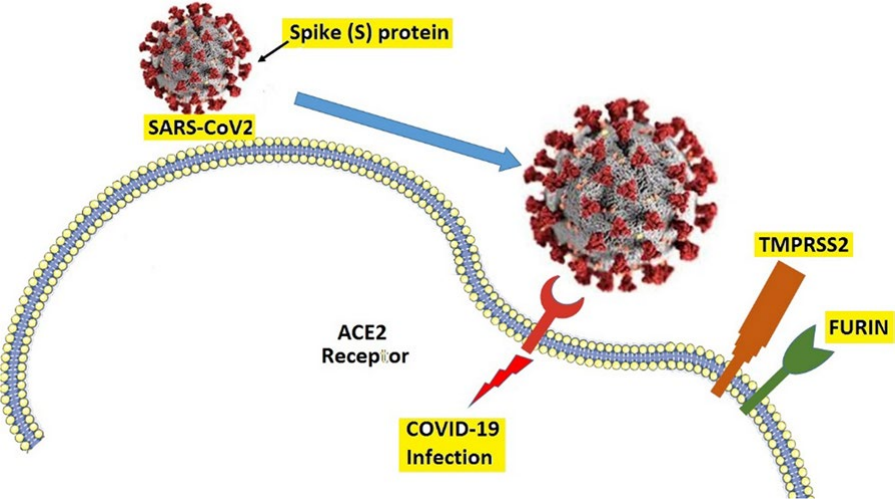
The role of spike protein and ACE2 receptor in the internalization of SARCE-CoV2. The transmembrane proteases of TMPRSS2 and FURIN 2 facilitate viral infection

Among the structural proteins, glycoprotein S or spike protein, which abundantly covers the surface of SARS-CoV-2, binds to the host cell receptor of angiotensin-converting enzyme 2 (ACE2). It is the major contributor to virus entry into the cell and causing infection [3, 4]. Notably, SARS-CoV-2 does not use other coronavirus receptors such as aminopeptidase N and dipeptidyl peptidase. By binding S protein to the receptor, transmembrane protease/serine subfamily member 2 (TMPRSS2), a known human protease located primarily in the airways and alveolar cell membranes, activates and facilitates virus entry into the cell by cleaving protein S and ACE2. Furin is another enzyme found in the host cell which has a crucial role in the entrance of the virus (Fig. 1) [5, 6]. In addition to TMPRSS2 and furin, other S-activating proteases including cathepsin L, trypsin, elastase, thermolysin, factor Xa, and plasmin may also have a role as SARS-CoV-2 entry cofactors. In the lack of prior ACE2 binding, these proteases can excessively proteolyze S proteins and inactivate virus infection [4, 7]. Thus, SARS causes more severe clinical signs and symptoms in tissues with high ACE2 densities on the cell surface, especially vascular endothelial cells of the lung and extrapulmonary tissues such as the gastrointestinal tract, kidneys, liver, testes, and heart [4].

The natural role of ACE2 is to reduce the concentration of tissue angiotensin II (Ang II) by its conversion to Ang-(1-7), which acts as an anti-inflammatory agent. The major pathologic aspect of COVID-19 is the elevation of Ang II levels directly related to viral load and lung damage. Ang II promotes the expression and activation of inflammatory cytokines such as interleukin (IL)-6 and thrombin formation and inhibition of fibrinolysis. SARS-CoV-2 also has a strong affinity to bind to porphyrins and reduces the level of functional intracellular hemoproteins [4, 8]. Dysfunction of porphyrins and hemoproteins leads to the release of free iron, especially from dying cells, which results in a severe systemic inflammatory response, tissue damage and a pro-thrombotic state [8]. In this situation, the alveolar epithelial barrier is destructed, the lung defense mechanism is weakened and the permeability of the pulmonary arteries increases. It leads to recruitment and influx of activated neutrophils and macrophages, pulmonary edema, and eventually acute respiratory distress syndrome (ARDS). The alveoli infiltrated with activated neutrophils, cytokines/chemokines, and protein-rich secretions would not ventilate enough to provide oxygen. Therefore, some factors by which to strengthen the immune system may help potentialy control COVID-19 and related respiratoty symptoms [8].

At present, there is no assured treatment that can directly target SARSE-CoV2. Moreover, variable genetic mutations and the emergence of more resistant strains of the virus also calls into question the efficacy of designed vaccines. However, several adjuvant therapies can be used to control the symptoms and severity of COVID-19 until discovering an anti- SARSE-CoV2 drug in the future. Some vitamins, minerals and herbal derivatives are efficient supplementals in maintaining the function of the immune system and have synergistic effects next to medical therapies [9, 10]. Previous studies have shown an association between some vitamin and mineral deficiencies and immunodeficiency against respiratory pathogens, as well as more extended recovery periods from disease. For example, it is evidenced that C, D and E vitamins and some minerals like zinc, selenium, and omega-3 fatty acids enrich the immune system to prevent and treat infectious diseases. It has been claimed that concomitant use of these compounds with azithromycin may be more effective in the rapid recovery of a patient infected by SARS-CoV-2 [11]. Interferons (IFNs) are internal antiviral agents of which, in combination with such supplements, may have synergistic treatment effects on the COVID-19 disease [12, 13]. Hereby, this article aims to discuss the anti-inflammatory effects of IFNs on SARS-CoV-2 infection and rather complementary effects of vitamin D and minerals.

## The common pattern of virus-induced hyperinflammation of respiratory viruses

The twenty-first century has a global impact on health due to the spread of a variety of respiratory viruses following successive mutations, which cause acute inflammation with high mortality rates. Overproduction of inflammatory cytokines in various SARS-CoV-2 viral infections indicates a severe immunopathological process [14, 15]. Due to the manifestations of influenza infection, including high levels of cytokines and chemokines such as IL-6, IL-1β, TNF-α, IL-10, and elevated serum ferritin levels, a similar association was shown between infection caused by the SARS-CoV-2 viral epidemic and seasonal human influenza viruses [16]. In influenza and COVID-19 infections, cytokine storm is closely related to coagulopathy and disseminated intravascular coagulation [17]. Both influenza and SARS-CoV viruses induce NLRP3 (NLR family pyrin domain containing 3) inflammasome activation [18], associated with pyroptosis—a highly inflammatory form of lytic programmed cell death- upon infection with intracellular pathogens. Lymphocytopenia, as well as reduced polyfunctionality and cytotoxicity of T-cells and NK cells due to the continuous expression of inhibitory markers such as programmed cell death protein-1 (PD-1) and T-cell immunoglobulin domain and mucin domain protein-3 (TIM-3), are characteristics of both influenza infection and COVID-19 [19, 20]. The reverse correlation between PD-1 and TIM-3 protein markers with total counts of CD8- and CD4-T cells, but not neutrophil counts, makes both parameters a good predictive criterion for COVID-19 progression and severity [20]. TIM-3 participates in cytokine storm during COVID-19 by activating infected macrophages and negatively regulating the Th1 immune response in the cytokine storm, and subsequently causes overstimulation of the innate immune system [20]. In addition to TIM-3, the activation of the PD-1/PD-L1 pathway in severe H1N1 influenza A infection has been demonstrated in tissue samples of the lower respiratory tract in pediatric patients and their dendritic and T cells as well [21, 22]. PD-L1 expression levels are inversely related to the number of CD8 + T cells in these patients, and inhibition of this pathway improves the number and function of CD8 + T cells [23]. Rutigliano et al. showed that decreased CD8 + T cells activity in influenza A virus infection in mice was associated with increased PD-1 expression [24]. They found that blocking PD-L1 in vivo can reduce the virus titer and increases the number of CD8 + T cells but not their activity. Another study reported that the recovery period from influenza infection in PD-1 -/- mice are much longer than the wild ones [25]. These findings show the dual role of the PD-1 / PD-L1 pathway, which negatively regulates CD8 + T cells and slows virus clearance.

As mentioned, severe cases of influenza and COVID-19 share a similar immune response, including a reduced number of circulating CD8 + and CD4 + T cells and increased amounts of proinflammatory cytokines [26, 27]. The lower number of acute immune cells in the acute phase of severe disease may be due to the migration of these cells to the respiratory tract; in fact, there may be no reduction in the production of immune cells. Autopsy of patients with COVID-19 showed diffuse infiltration of lymphocytes, especially CD8 + T cells into the lungs, along with focal infiltration into the liver, kidney, pancreas, intestine, adrenal, and pericardium. Such lymphocyte migrations and following cytokine storm could promote apoptosis or necrosis of T cells and consequently reduce their number in blood circulation [28].

## Interventions of IFNs and their agonists with SARS-CoV-2 infection

The cytokine storm, an abrupt rise of serum inflammatory cytokines and chemokines in SARS-CoV-2, influenza, and MERS-CoV infections trigger a severe systemic inflammatory response that must be controlled to limit tissue damage [16]. Interferon cytokines as the first line of defense against viral infections are secreted by immune-activated cells and activate natural killer cells (NK) and macrophages. Type I IFNs includes IFN-α and IFN-β, while IFN-γ and IFN-λ belong to type II and type III IFNs, respectively [29]. IFNs bind to their receptors on the cell surface and activate several genes involved in the antiviral process by inducing the Janus-activated kinase (JAK)/signal transducer and activator of transcription (STAT) signaling pathway. In this signaling pathway, the activated IFN-receptor induces Janus kinase 1 (JAK1) and Tyrosine kinase 2 (TYK2), which then phosphorylate STAT1and STAT2. These phosphorylated factors enter the nucleus and are assembled with interferon-regulatory factor 9 (IRF9). The activated IRF9 stimulates interferon-stimulated gene factor 3 (ISGF3) and subsequently the transcription of interferon-stimulated genes (ISGs). ISGs are important contributors to virus-induced immune responses [30, 31].

Anti-inflammatory effects are another role of IFNs that are associated with the suppression of pro-inflammatory cytokines such as IL-1, IL-18 and IL-12 and the induction of anti-inflammatory cytokine IL 10. In SARS-CoV-2 infection, there are abnormally low levels of antiviral cytokines, especially type I IFNs [17, 18]. Therefore, IFNs are considered an important target to control cytokine storms and inflammation within the treatment of COVID-19. A well-documented strategy of coronavirus reported as an elimination of host interferon's defense system through interference to their production and signaling pathway. For example, a reduction in IFN-γ expression has been observed in CD_4+_ T cells of patients with COVID-19 associated with disease severity [19]. IL-6 can also differentiate Th2 cells from Th0 by activating the STAT3 signaling pathway and eventually producing Th2 cytokines such as IL-13 and IL-4, as well as suppress cytokine signaling 1 (SOCS-1). SOCS-1, as an inhibitory molecule through its effects on STAT1 phosphorylation, can disrupt the production of IFN-γ and IL-2 and lead to a decrease in the level of these cytokines by Th1 cells [32]. On the other hand, increasing IL-6 and finally SOCS-1 by interfering with STAT4 phosphorylation has an inhibitory effect on IFN-γ and IFN-II production. These IFNs are involved in the cytolysis of infected cells by stimulating and activating killer cells, including NK and CD8 + -T cells. One of the main mechanisms in the removal of virus-infected cells in the progression of apoptotic pathways and their associated molecules by pro-apoptotic molecules such as granzyme B, which are produced and secreted by killer cells [33]. According to the described mechanism, the survival of infected cells can be affected by IL-6 because this multifunctional cytokine can induce anti-apoptotic molecules by stimulating Th17 differentiation and IL-17 production [33]. Another chosen mechanism by the virus for the development is the cooperation of IL-6 and IFN-I. These cytokines increase the survival of the infected cell by increasing inhibitory molecules such as PD-L1 (CD274) on the surface of the infected cell. The binding of PDL-1 to PD-1 (CD279) on CD8 + -T cells prevents apoptosis induced by these cells [33, 34].

Coronaviruses also avoid interactions with pattern-recognition proteins (PRPs) responsible for inducing pro-inflammatory reactions and antiviral responses mediated by IFN [20]. These mechanisms ultimately interfere with the production of IFNs and cause a delayed antiviral response mediated by IFNs. Some in vitro studies have shown that IFN- therapies can inhibit viral replication and the combination of IFNs has a synergistic effect in this regard [35]. Thus, IFN- therapy is an accepted treatment strategy to induce antiviral immune responses. However, IFN administration in the early stages of infection seems to affect effectively, leading to devastating responses in severe or later stages of the disease [36]. Although antibiotics are known as antibacterial agents, some also have antiviral effects, such as Macrolides. Macrolides composed of a large lactone ring bind to the 50S subunit of the bacterial ribosome and interfere with protein synthesis [37]. There are some indications of macrolides to relieve viral respiratory infections [21]. The antiviral activity is attributed to their binding to IFN-receptor and inducing STAT1/2, IRF7, IRF9, and production of ISGF3 as well [22]. Clarithromycin and Leucomycin are the examples of Macrolides, used against the influenza virus to increase IFN-α production [23, 38]. Azithromycin is another macrolide that inhibits rhinoviruses via potentiating IFN-I signaling. It activates IκB-kinase (IKK), IKK-ι/ε, and TANK-binding kinase 1 (TBK-1) signaling pathway, which stimulates the IRF factor, and IL-28 and IL-29 receptors [39, 40]. Azithromycin can induce the gene expression of IFN-β and IFNλ1, toll-like receptor 3 (TLR3), melanoma differentiation-associated protein 5 (MDA5), RIG-I-like helicase, and retinoic inducible gene I (RIG-I) in bronchial epithelial cells [40, 41]. The aforementioned agents act as antiviral proteins in rder to reduce the viral load. Azithromycin also improves the cell sensitivity to viral infections through upregulation of pathogen recognition receptors (PRRs) of IFIH1, DDX58, and ISGs including IFITM3, MX1, and RASD2 [42, 43]. In addition to ACE2, CD147 is another binding receptor, that internalizes SARS-CoV-2 virus. Azithromycin may interfere with CD147 and the virus interaction process.

Ribavirin (Virazole), a guanosine analog, is another drug introduced by Witkowski et al. and originally used only to treat severe respiratory syncytial virus (RSV) infection in children [44]. It has a wide range of functions against RNA and DNA viruses, including infection with Lhasa fever virus, influenza A and B, and other viruses. It is also effective in combination with IFN-α in the treatment of chronic hepatitis C infections [45, 46]. It combines with viral RNA and, in addition to inhibiting the normal viral replication, causes mutations in its genome. It also inhibits RNA-dependent RNA polymerase activity. Following their bioinformatics studies, it has been claimed that the drug could bind to the active site of the enzyme and, by inhibiting it, could be a potent inhibitor for SARS-CoV-2 infection [47, 48].

Studies have indicated that some nutrients, including vitamin D, magnesium and zinc, play an essential role in the immune system and modulate the IFN signaling pathway. Adequate amounts of such micronutrients are crucial to ensure the proper functioning of the immune system. Vitamin D can control the production of proinflammatory cytokines and the cytokine storm observed in COVID-19 through affecting the nuclear factor-kB and other related pathways. Magnesium is a critical cofactor in the synthesis and activation of vitamin D [49]. Zinc is also important for the development of immune and other cells, so its deficiency leads to defects in humoral and cellular immunity [50].

## Potential role of activation of the vitamin D in acute respiratory distress syndrome (ARDS)

Vitamin D **(**VitD), as one of the oldest evolutionary hormones with a known role in the absorption of calcium from the intestine, plays a role in regulating the immune system [51]. VitD receptor (VDR) is detected in all immune cells especially monocytes and macrophages and regulates their activity. it can potentially prevent a cytokine storm by reducing the expression of pro-inflammatory cytokines. Studies have shown a correlation between VitD deficiency and an increased risk of upper respiratory tract infection. A meta-analysis in 2019, using approximately 10,000 individual participant data from 25 RCTs, concluded that vitamin D supplementation could reduce the risk of upper respiratory infections by 19%. The idea comes to the question is whether this conclusion also applies to coronavirus infections [52].

**Fig. 2:**
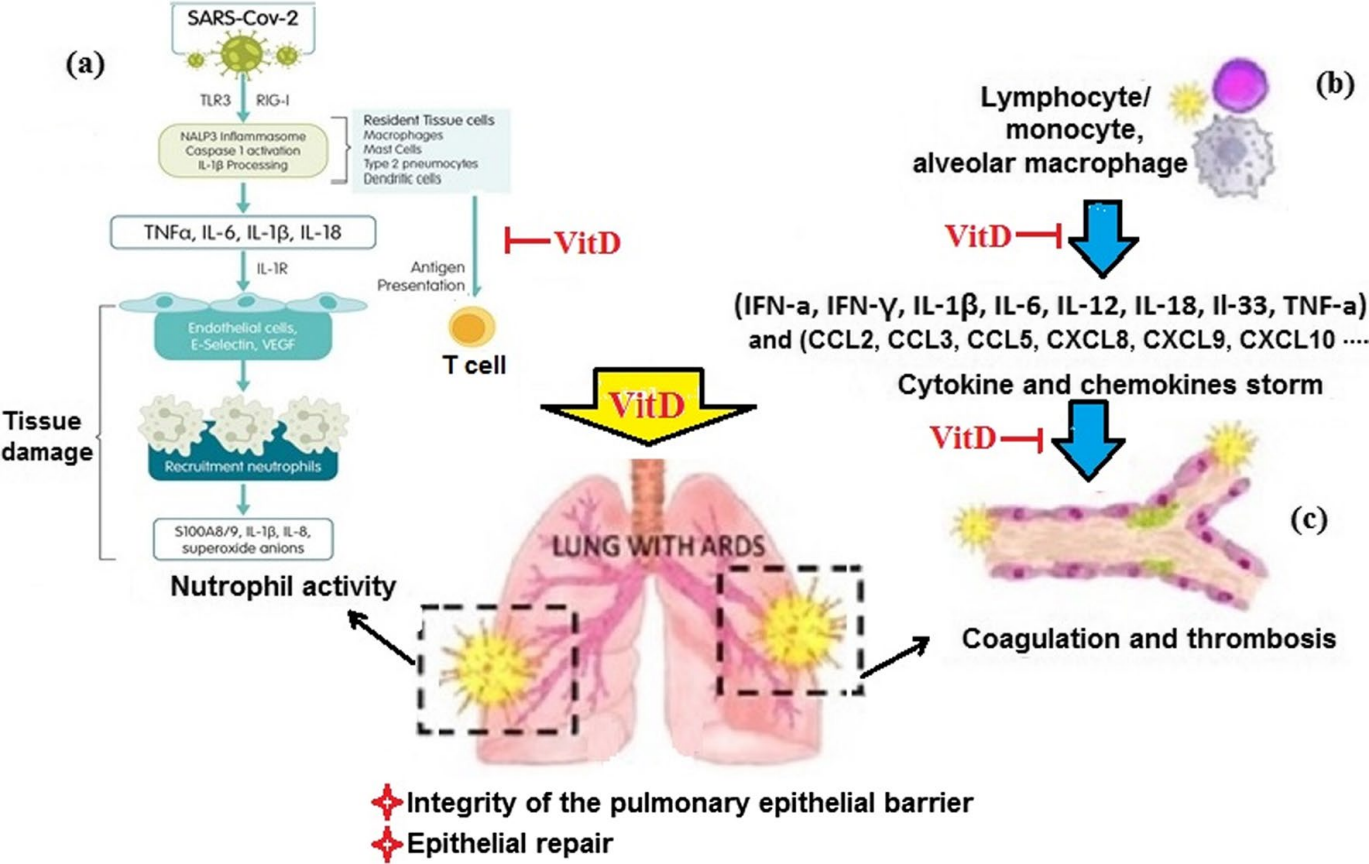
**a** recruitment of activated neutrophils into the inflamed interstitium and alveolar space by cytokines and chemokines secreted from infected epithelial and endothelial cells and activated alveolar macrophages; **b** cytokines and chemokines secreted by activated leukocytes cause cytokine and chemokine storms that are involved in the pathogenesis of ARDS; **C** dysregulation in homeostasis resulted in intra-alveolar or systemic fibrin clots and thrombotic complications. In each mentioned complication, the positive effects of VitD on the prevention and improvement of viral infections have been demonstrated

As shown in Fig. 2, the signaling pathway activated through VitD complexed with its receptor (VDR) reduces cytokine and chemokine storm, modulates neutrophil activity, regulates the renin-angiotensin system (RAS), maintains pulmonary epithelial barrier integrity, and stimulates epithelial repair [53]. Animal studies and in vitro models have detected high levels of VDR in the alveolar type II cells (ACII) of the lung. They have shown that VitD acts like an endocrine system by increasing the production and secretion of cytokines such as IL-1β, IL-6, IL-12, IL-18, IL-33, IFN-α, IFN-γ, TNF-α, and chemokines such as CCL2, CCL3, CCL5, CXCL8, CXCL9, CXCL10 in acute respiratory syndrome [54, 55]. For example, following LPS treatment, VDR-knockout mice had more severe ARDS than wild-type mice. Xu et al., in their study on the mechanism of VitD effect on acute lung injury (ALI) induced by lipopolysaccharide (LPS) reported that VitD may attenuate LPS-induced ALI by modulating the expression of the RAS components, including ACE, ACE2, renin and Ang II. Li et al., suggested that VitD is a negative endocrine regulator of the RAS and inhibits renin biosynthesis [56]. Ishii showed that inhibition of renin receptors reduces inflammatory symptoms, including interstitial edema, neutrophil count, hemorrhage, and the amount of pro-renin in the lung tissues of the study model [57]. Takano et al. suggested that the inhibitory effects of VitD on neutrophil recruitment in the hamster model of ALI could be due to its suppressive effect on the interleukin-8 (IL-8) gene involved in the pathogenesis of ALI [58].

Some genes regulated by VitD are key genes in the immune system's response to many bacterial and viral infections [59]. Cathelicidin antimicrobial peptides (CAMP) of LL-37 and FALL-39 are antimicrobial molecules that are primarily synthesized and stored in the lysosomes of polymorphonuclear cells, and macrophages after activation by viruses, bacteria, parasites, fungi. VitD also stimulates the transcription of cathelicidins [60, 61]. The activated VDR by VitD acts as a transcription factor that influences the transcription of several genes including the hCAP18 gene that encodes cathelicidin. Following cleavage by proteinase 3, the peptide precursor CAP-18 (18 kDa) is processed into active forms LL-37 and FALL-39 [62, 63].

The mechanism of action of cathelicidins is that, following the phagocytosis of the invasive agent and the attachment of the phagosomes to the lysosomes, the cathelicidins lyse it by damaging and perforating the membrane of the invasive agent cell, and providing the conditions for its complete destruction through lysosomal degrading enzymes. Cathelicidins are also able to destroy the cover of enveloped viruses such as those of the Coronavirus family [64]. Thus, vitamin D can help regulate the immune system response and clear the SARS-CoV-2 virus by producing LL37. Remarkably, exposure to fine particulate air pollution, dust, and cigarette smoke can worsen the severity of COVID-19 via reducing effective immune response, modulated by LL37, and interfering with LL37 in the destruction of viruses [65]. Therefore, the use of vitamin D supplements in areas or large cities with air pollution problems can be helpful in the treatment of COVID-19 disease.

**Fig. 3:**
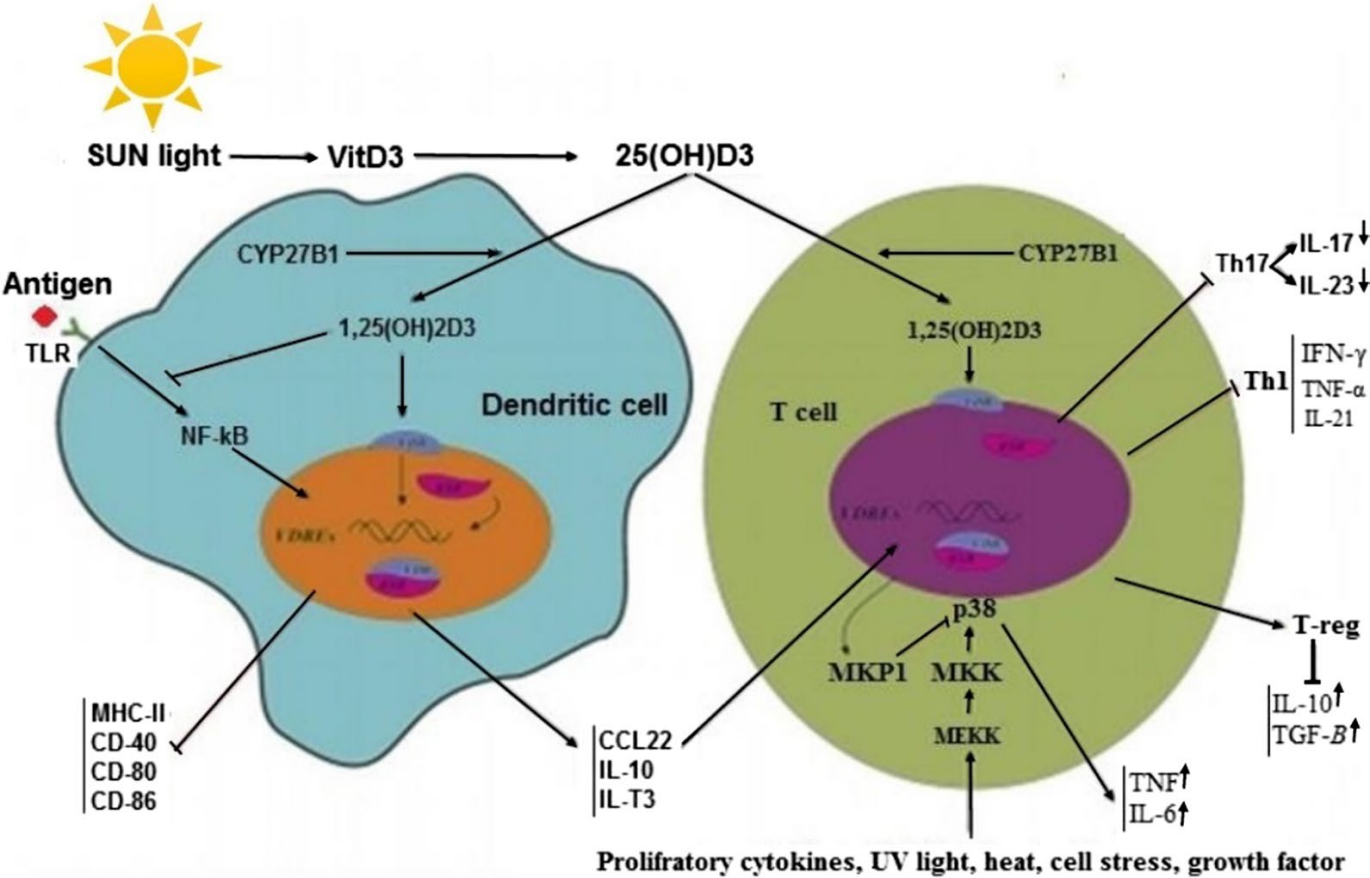
Primary mechanisms by which VitD regulates the function of dendritic cells and T lymphocytes. In DCs, VitD binds to the VitD-R, which is complexed with RXR in the nucleus. VDR/RXR complex decreases expression of MHC-II, CD40, CD80, and CD86 and enhances the expression of CCL22, IL-10, and IL-T3, which results in the induction of T-cells. The VitD stimulates TCR, which induces VDR expression via the alternative p38 MAPK pathway. Then, VitD binding to VDR leads to inhibition of proinflammatory cytokine expression, including IFN-γ, IL-17, and IL-21, and the development of T-reg cells. *DC* dendritic cell, *MHC* major histocompatibility complex, *CCL22* chemokine (C–C motif) ligand 22, *MAPK* mitogen-activated protein kinase, *T-reg cell* T regulatory cell

Figure 3 shows the possible inhibitory mechanism by VitD to prevent cytokine and chemokine storms by regulating the function of both dendritic cells (DCs) and T cells. In DCs, VitD/VDR complex in the nucleus down-regulates the expression of CD40, CD80, CD86, MHC-II, and up-regulates the expression of IL-3, IL-10 and CCL22, which results in the induction of T cells [66, 67]. VitD/VDR complex also can control TLRs signaling through inhibition of miR-155-SOCS1 pathway and decrease in the pro-inflammatory cytokine production such as TNF-a, IL-6, and IFN-γ [68]. In T-cells, VitD/VDR can make a complex with RXR that leads to induction of MKP1 and inhibition of the p38-MAP kinase pathway [69]. By inhibiting the MAP kinase pathway, p38 is dephosphorylated, which down-regulates the pro-inflammatory cytokines such as IFN-γ, IL-6, Il-17, IL-23 and TNF-α. Dephosphorylated p38 can also inhibit the maturation of DCs, and the differentiation of Th1 and Th17 [69, 70].

Nowadays, VitD deficiency is considered a global health problem. Studies have shown a significant association between VitD deficiency and the progression of asthma attacks, which may threaten the persistence of infection and sepsis [71]. A systematic review and meta-analysis examined the efficacy of high-dose VitD in pediatric asthma and showed that high-dose VitD may prevent asthma exacerbations [72]. Also, Jae Hyoung Im and et al. showed vitamin D deficiency with a deficiency (< 20 ng/dl) in 76% of the SARS-COV-2 infected patients and a severe deficiency (< 10 ng/dl) in 24% [73]. Thus, these studies again confirm the potential positive effects of VitD supplementation in preventing the severity of COVID-19.

## Magnesium effect on COVID-19 through VitD

Some Studies have established a relation between magnesium deficiency, decreased immune cell activity, and increased inflammation, which could be significant in COVID-19-associated cytokine storm pathology [74]. Magnesium, like other trace elements, acts mainly through its cofactor or structural role for enzymes. Enzymes containing cytochrome P450 are examples of these enzymes, which play a role in the metabolism of VitD. They are involved in both the activation and inactivation of VitD [49, 75]. The activation is mediated by 25-hydroxylase (CYP2R1) and 1α-hydroxylase (i.e., CYP27B1), and deactivation is catalyzed by 24-hydroxylase (CYP24A1)[76]. 25-hydroxylation and synthesize of 25(OH)D from VitD_3_ or VitD_2_ is occurred in the liver and followed by 1α-hydroxylation of 25(OH)D to active 1,25(OH)_2_D in the kidney. Both 25(OH)D and 1,25(OH)_2_D are metabolized to inactive forms of 24,25-dihydroxyvitamin D and 1,24,25-trihydroxyvitamin D by 24-Hydroxylase, respectively [77]. The dependence of these enzymes on magnesium may indicate the role of magnesium in maintaining active levels of VitD, and in controlling the severity of COVID-19 disease, while it requires more clinical and experimental investigations.

## The roles of Zinc in the inhibition of acute respiratory distress syndrome (ARDS)

Zinc, an important micronutrient especially for enzyme activity and zinc fingers, is essential for regulating both innate and adaptive immune systems and maintaining immune tolerance. It holds the proliferation and maturation of leukocytes and lymphocytes and modulates the inflammatory responses. Several studies have shown the association between zinc deficiency and the prevalence of respiratory infections among the population. Rerk suppaphol et al., in a double-blind placebo study of zinc supplementation in the treatment of acute respiratory tract infections, showed a 45% reduction in the rate of acute respiratory infections [78]. Singh et al., in their randomized, double-blind, placebo-controlled trial study, reported the ameliorative effect of zinc in reducing the duration of colds [79]. Some other studies have indicated the antiviral activity of zinc against various viruses including influenza [80], rhinovirus, herpes virus, respiratory syncytial virus and transmissible gastroenteritis virus [81]. Such immunomodulatory and antiviral properties of zinc, highlights its potential role as a supportive agent in the treatment of COVID-19.

Zinc deficiency has a significant effect on bone marrow and reduces the production of B lymphocytes and T lymphocytes following a decrease in the number of immune progenitor cells [82]. Its deficiency also alters the function and number of blood polymorphonuclear, NK cells, and lymphocytes, especially T cells [81]. Zinc is a major component of the hormone thymolin, which is involved in the development of T cells in the thymus gland [83]. It is an essential factor involved in complement activation and helps regulate cytokine secretion such as IL-2, IL-6 and TNF by reducing the formation of pro-inflammatory Th9 and Th17 cells [84]. Zinc induces differentiation of monocytes to macrophages, increases the phagocytic potency of macrophages, and stimulates them to produce IL-12 to activate NK and T cells. Zinc also upregulates the production of IL-2, IFN‑α and IFN-γ and downregulates the production of IL-10 resulting in to promotion of antiviral reactions. On the other hand, IFNs can stimulate the influx of zinc into the target cells. Decreased levels of IL-10 positively affect macrophage function and Th1 response [81, 85, 86]. IFNα antiviral activity is mediated through JAK1/STAT1 downstream signaling and upregulation of antiviral enzymes, including protein kinase RNA‑activated (PKR) and latent ribonuclease (RNaseL) [87]. Such antiviral enzymes are involved in the degradation of viral RNA and inhibition of viral RNA translation. Both IFN-γ and IL-12 also play a crucial role in the destruction of various pathogens through a mechanism including downregulation of ERK1/2 and NF‑κB pathways [88]. Regulation of ERK1/2 and NF‑κB pathways has been shown to be necessary for the protective effect of zinc on the lungs in the infection states. Low zinc status upregulates IKK activity and subsequent NF‑κB signaling resulting in upregulation of target genes of TNFα, IL‑1β, and ICAM‑1 [89, 90]. In their study of primary human lung cells, Bao et al. reported that in the absence of zinc, treatment with IFN-γ and TNF-α, as well as activation of Fas-R signaling, would lead to cell apoptosis and impaired pulmonary epithelial barrier function [91]. Aydemir et al. also showed that zinc regulates IFN-γ expression in human activated T lymphocytes isolated from individuals supplemented with 15 mg/day zinc [92]. The upregulated IFN-γ in activated human T lymphocytes, reduces the release of the cytokine. Such overall results indicate that zinc is a vital factor in the protection of pulmonary epithelium against acute damage.

## Conclusions

COVID-19, as a potentially life-threatening disease, has received serious attention from researchers using various treatment strategies. Targeted treatments against cytokines can prevent the cytokine storm, which brings the disease to its final stage. VitD, by affecting NF-κB and other pathways, can attenuate various pro-inflammatory cytokines involved in the cytokine storms. Magnesium, the critical element in the synthesis and activation of VitD, acts as a cofactor for many enzymes involved in VitD metabolism. Low zinc status impairs immune response and increases susceptibility to viral, bacterial, and fungal infections. Excessive inflammatory response overproduces pro-inflammatory cytokines and cytokine storm, which play a significant role in COVID‑19 pathogenesis. Therefore, it seems that increasing zinc intake may be effective in the treatment of COVID-19 by reducing viral infection and preventing ARDS. So, it can be concluded that concomitant use of a standard drug with VitD, magnesium, and zinc may effectively control COVID 19 in the early stages and reduce mortality.

## Data Availability

Data presented in this manuscript is available upon request.

## Abbreviations

ACE2: Angiotensin-converting enzyme 2
ARDS: Acute respiratory distress syndrome
COVID-19: Coronavirus disease-19
DCs: Dendritic cells
IFN: Interferon
IL: Interleukin
JAK: Janus activated kinase
STAT: Signal transducer and activator of transcription
SARS-CoV-2: Severe acute respiratory syndrome coronavirus 2
TMPRSS2: Transmembrane protease/serine subfamily member 2
VitD: Vitamin D
